# Statistical regularities modulate attentional capture independent of search strategy

**DOI:** 10.3758/s13414-018-1562-3

**Published:** 2018-07-02

**Authors:** Benchi Wang, Jan Theeuwes

**Affiliations:** 1 Department of Experimental and Applied Psychology, Vrije Universiteit Amsterdam, Van der Boechorststraat 1, 1081 BT Amsterdam, The Netherlands; 2 Institute Brain and Behavior Amsterdam (iBBA), Amsterdam, The Netherlands

**Keywords:** Attentional capture, Suppression, Feature search mode, Statistical regularities

## Abstract

An earlier study using the additional singleton task showed that statistical regularities regarding the distractor location can cause an attentional bias that affects the amount of attentional capture by distractors and the efficiency of selection of targets. The distractor singleton was systematically present more often in one location than in all other locations. The present study investigated whether this bias also occurs when observers adopt a feature search mode, i.e., when they search for a specific feature (circle) between elements with different shapes, while ignoring a colored distractor singleton. It is assumed that in feature search, observers can ignore distractors in a top-down way and as such one expects that statistical regularities about the distractor location should not play a role. Contrary to this prediction, we found that even in feature search, both attentional capture by the distractors and the efficiency of selecting the target were impacted by these statistical regularities. Moreover, statistical regularities regarding the feature value of the distractor (its color) had no effect on the amount of capture or the efficiency of selection. We claim that statistical regularities cause passive lingering biases of attention such that on the priority map, the location containing a high probability distractor competes less for attention than locations that are less likely to contain distractors.

## Introduction

In everyday life, we constantly look around and use our visual input to guide our behavior. We intentionally search for our favorite coffee in the supermarket or look out for a specific color when searching for our car in the parking lot. In order to fulfill our needs, we often search with a particular top-down goal in mind that guides our search behavior. When searching for particular objects, we may sometimes experience that we attend to things in our environment for which we had no intention to look for. We may inadvertently attend to the road worker wearing a fluorescent orange safety jacket, a moving billboard along the roadside, or a waving hand in the crowd.

Attentional control has been considered to be the result of the above-described interplay between voluntary, top-down, goal-driven control and automatic, bottom-up, stimulus-driven control (for reviews, see Burnham, 2007; Corbetta & Shulman, 2002; Theeuwes, 2010, in press). Recently, however, it was argued that this classic theoretical dichotomy may no longer hold as often selection cannot be explained by current selection goals or the properties of the environment (Awh, Belopolsky, & Theeuwes, 2012; Failing & Theeuwes, 2017). Awh et al. (2012) suggested that a third category labelled as selection history signifying that the history of attentional deployments can elicit lingering selection biases competing for selection, that are unrelated to top-down goals or the physical salience of items.

One way the history of attentional deployments elicits lingering selection biases is when particular statistical regularities are present in the display. Several studies have demonstrated that statistical regularities can bias selection. For example, the efficiency of searching for a target can be improved when the target consistently appears in specific locations adopted in previously seen displays relative to random locations (Chun & Jiang, 1999). Moreover, Geng and Behrmann (2005) showed that targets present in high probability locations are detected faster than those in low probability locations (see also Jiang, Swallow, Rosenbaum, & Herzig, 2013). In these studies, in which the target is likely to appear in one particular location, it may not be surprising that there are benefits in target selection when the target is *immediately relevant* for the task. In order to find the target, participants need to direct their attention to potential target locations, which makes it likely that participants either implicitly or explicitly pick up on the statistical regularities.

In a recent study, however, using the classic additional singleton task, Wang and Theeuwes (2018a, b) demonstrated how statistical regularities can bias selection but with respect to the distractor location. Participants searched for a salient shape singleton (i.e., a diamond between circles or a circle between diamonds) while ignoring a colored distractor singleton. The colored distractor singleton was systematically present more often in one location than in all other locations. The results showed for this high probability location: (1) less efficient selection of the target and (2) a reduction in the amount of attentional capture by distractors. Crucially, they reported a spatial gradient from this high probability location as the attentional capture effect scaled with the distance from this location. Experiment 2 showed that most observers were not aware of the statistical regularities present in the display. These findings were interpreted as evidence that implicit statistical regularities that cannot be reported by the observer, can bias attention such that locations that have a high probability of containing a distractor compete less for attention than all other locations (see also Ferrante et al., 2017; Goschy, Bakos, Müller, & Zehetleitner, 2014). Specifically, it was argued that the location that was highly likely to contain a distractor was suppressed relatively to all other locations as there was a spatial gradient of suppression surrounding the location (Mounts, 2000). It is crucial to stress that in Wang and Theeuwes (2018a, b), statistical regularities regarding distractors – which are *irrelevant* for the task – dramatically modulated visual selection, indicating that people can not only learn from attending relevant items but also from ignoring items that are irrelevant.

Wang and Theeuwes (2018a, b) showed that there was less capture for distractors present at the high probability location relative to all other locations; yet relative to the no-distractor condition there was still substantial capture for distractors at the high probability location. One might question that the suppression observed in this previous study may only be related to the fact that participants searched for a unique shape singleton (i.e., a circle between a diamond or a diamond between circles) instead of a specific target (e.g., a circle between diamond, triangle, and square). Searching for a unique shape singleton has been labelled as the “singleton detection” mode, in which observers can adopt a strategy to search for a discontinuity (Bacon & Egeth, 1994), and then the colored distractor singleton (which is also a discontinuity) captures attention. If observers no longer search for a unique shape singleton but instead search for a specific target between several different shapes, they strategically choose the so-called feature search mode. In this mode, there is no or less measurable capture by the irrelevant distractor (Bacon & Egeth, 1994; Lamy, Leber, & Egeth, 2004; Leber & Egeth, 2006; but see Theeuwes, 2004).

The present study tested whether the strategic use of a feature search mode would eliminate the benefit provided by the statistical regularities. The general consensus is that when the feature search mode is adopted, participants are able to impose top-down selectivity which should reduce or even eliminate capture by stimuli that do not match the attentional set (e.g., Leber & Egeth, 2006). In other words, top-down control should prevent capture by the irrelevant salient singleton (Bacon & Egeth, 1994). It is assumed that due to this “feature-search” top-down set, participants completely ignore the salient distractor because its features do not match the feature participants are looking for (Bacon & Egeth, 1994; Leber & Egeth, 2006; Theeuwes, 2010). However, more recent studies investigating the neural basis of this ability to avoid attentional capture during feature search have revealed that capture can be avoided because this salient distractor is suppressed. Studies have demonstrated that in feature search, the irrelevant distractor singleton elicits a PD component of the event-related potentials (ERPs) signal, which is generally believed to be a neural maker of suppression (Feldmann-Wüstefeld, Uengoer, & Schubö, 2015; Hickey, Di Lollo, & McDonald, 2008; Sawaki & Luck, 2013). This implies that even in feature search, the irrelevant distractor singleton does have an effect on processing (i.e., it generates a PD), and as such it is likely that statistical regularities regarding the distractor may have an effect. It should be realized, however, that if the suppression of the salient distractor is strong enough, we may not observe an effect on attentional capture, i.e., capture may be fully eliminated. If the suppression is space-specific – and not feature-specific as for example assumed by Sawaki and Luck (2013) – we may, however, observe an effect when the target singleton happens to be presented at that location. We expect that target selection is less efficient when it is presented at that suppressed location, effects which we have observed in previous studies that involved singleton search (Wang & Theeuwes, 2018a, b).

## Experiment 1

We used displays in which observers searched for a unique circle among diamonds, triangles, and squares that would force observers to use a feature search mode. When in feature search, observers should be able to ignore the distractor completely (e.g., Bacon & Egeth, 1994) and therefore we expect no effect of the statistical regularities. If, however, feature search is not qualitatively different from a singleton detection mode (e.g., Theeuwes, 2004; Zehetleitner, Goschy, & Müller, 2012) we still expect to see an effect of the statistical regularities.

### Method

The study was approved by the Ethical Review Committee of the Faculty of Behavioral and Movement Sciences of the Vrije Universiteit Amsterdam.

#### Participants

Twenty-four adults (20 females, mean age: 20 years) were recruited for the present experiment with monetary compensation. Informed consent was signed by all participants before the study. They reported normal color vision and normal or corrected-to-normal visual acuity.

#### Apparatus and stimuli

Participants were tested in a dimly lit laboratory, and were required to hold their chin on a chinrest located 63 cm away from the 17-in. CRT monitor. An Eyelink® 1000 eye-tracker (1,000 Hz) was used to check fixation at the center. The stimulus presentation and response registration were controlled by custom scripts written in Python.

**Fig. 1.**
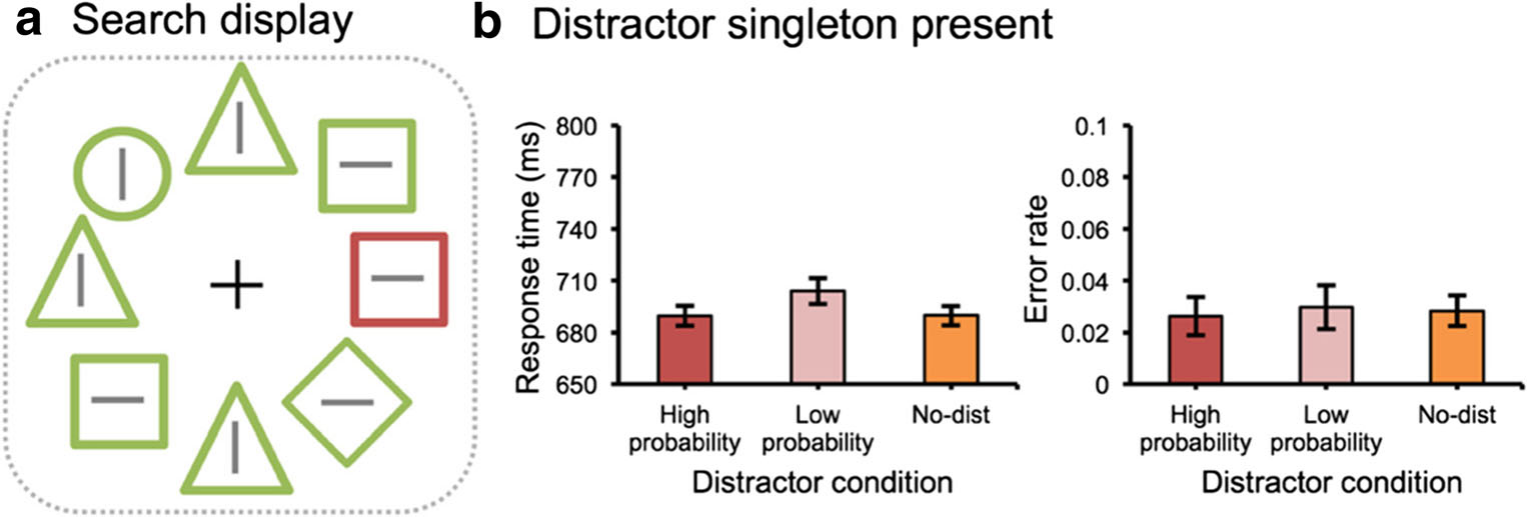
(**A**) The display setup and possible target and distractor locations. (**B**) The mean response etimes (RTs) (left panel) and the mean error rates (right panel) between different distractor conditions in Experiment 1. Error bars denote within-subjects 95% confidence intervals (Morey, 2008)

Eight discrete stimuli with different shapes (one circle, one unfilled diamond, three unfilled triangles, and three unfilled squares), each containing a vertical or horizontal gray line (0.15° × 1°) inside, created a visual search display (see Fig. 1A). These stimuli were present on an imaginary circle with a radius of 4°, centered at the fixation (a white cross measuring 1° × 1°), against a black background (17 cd/m^2^). The circle’s radius was 1°, and the other unfilled shapes were subtended 2° × 2°.

#### Procedure and design

Each trial started with a self-paced drift check, followed by a fixation cross that remained visible throughout the trial. After 500 ms, the search array was present for 3,000 ms or until response. Participants had to consistently search for the only circle in the display and indicate whether the line segment inside the target was vertical or horizontal, by pressing the “up” or “left” key as fast as possible with their dominant hand. The inter-trial interval (ITI) was randomly chosen to be from 500–750 ms.

The target was present in each trial with random color (red or green with an equal probability), and distractors had the same color as the target except that a uniquely colored distractor singleton (red or green with an equal probability, but different from the target color) was used in 66% of the trials. All conditions were randomized within each block. The possible location of the distractor singleton could be one of eight locations from the imaginary ring with a 4° radius. However, one of these locations had a high proportion of 65% (high probability location; i.e., 43% of the total trial number); and each of seven other locations had a low proportion of 5% (low probability locations; i.e., 3.3% of the total trial number). The high probability location was unchanged for each participant and was counterbalanced across participants. In the no-distractor condition, the target appeared at each location with equal chance. After 40 practice trials, six blocks each containing 120 trials were tested. Participants had to maintain fixation through the trial. If the participants’ gaze deviated more than 2° from fixation, if they did not respond, or if they pressed the wrong key, warning messages would be shown. Trials with larger gaze deviation were later re-tested in a random order until all trials were completed successfully.

### Results

Ten percent of trials with larger gaze deviation were later re-tested in a random order. Trials on which the response times (RTs) were larger or smaller than 2.5 standard deviations from the average RT per block per participant (3.8 %) were excluded from analyses.

#### Attentional capture effect

Mean RTs and mean error rates are presented in Fig. 1B. With *distractor condition* (high probability location, low probability location, and no-distractor) as a factor, mean RTs were entered into a repeated measures ANOVA and showed a main effect, *F*(2, 46) = 10.26, *p* < .001, partial *η*^*2*^ = .31. Subsequent planned comparisons showed that there was a significant attentional capture effect for a distractor present at low probability locations (low probability location vs. no-distractor), *t*(23) = 3.66, *p* = .001, *d* = 0.14, but not for a distractor present at the high probability location, *t* < 1 (high probability location vs. no-distractor). Importantly, however, the difference between the high and low probability location was reliable, *t*(23) = 3.51, *p* = .002, *d* = 0.14, suggesting that the attentional capture effect was eliminated for trials in which the distractor singleton appeared at the high probability location. There was no effect on error rates, *F* < 1.

#### Target at the high probability distractor location

**Fig. 2.**
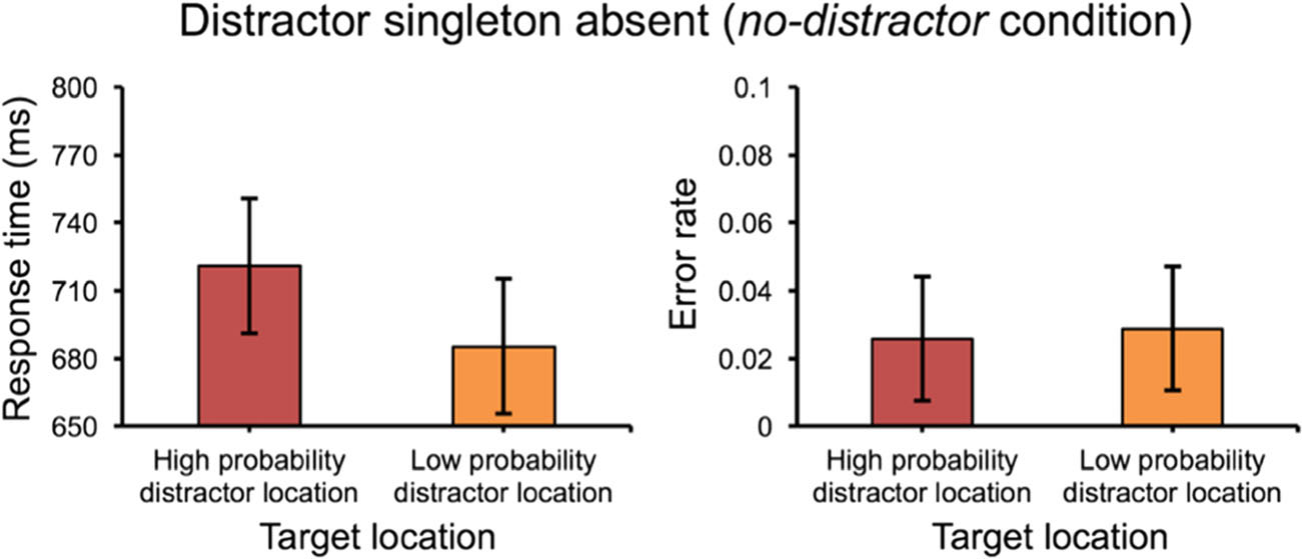
The mean response times (RTs) (left panel) and the mean error rates (right panel) in the no-distractor condition in Experiment 1. Error bars denote within-subjects 95% confidence intervals

To determine whether the efficiency of selecting the target was affected by the statistical regularities, we analyzed the RTs in the *no-distractor* condition for when the target happened to be present at the high relative to all low probability locations. Participants were slower when the target was present at the high probability distractor location than when it was present at low probability distractor locations, *t*(23) = 2.54, *p* = .018, *d* = 0.28 (see Fig. 2, left panel). There was no effect on error rates, *t* < 1 (see Fig. 2, right panel).

#### The spatial distribution of the suppression effect

**Fig. 3.**
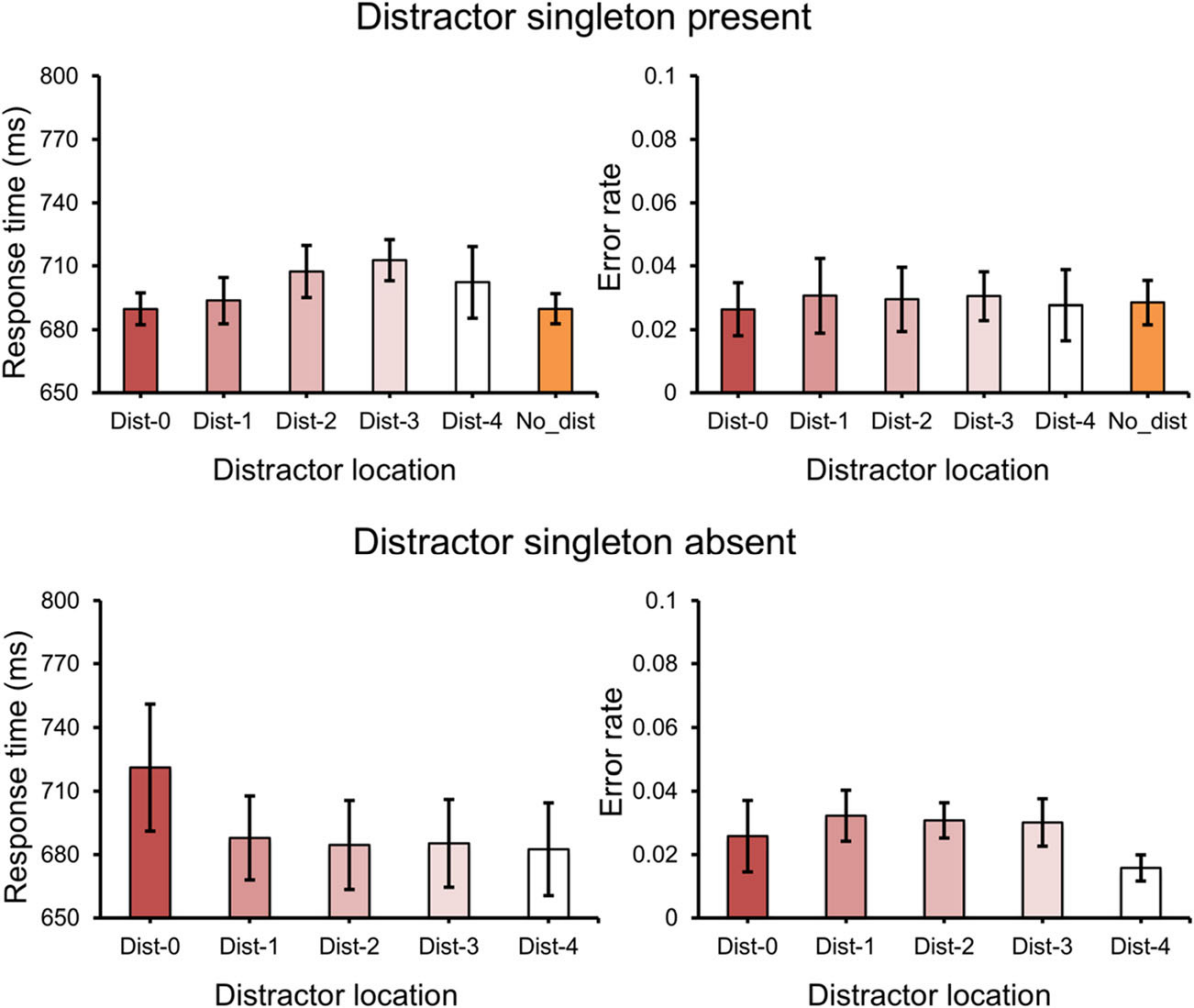
The spatial distribution of suppression effect by the means of response times (RTs) (left panel) and error rates (right panel) in Experiment 1. “Dist-0” means the high probability distractor location; “Dist-1” means the low probability distractor location with one unit (45° polar angle) away from the high probability distractor location, and so on. Error bars denote within-subjects 95% confidence intervals

RTs were examined when the distractor singleton appeared at different locations in relation to the distance from the high probability distractor location. If the suppression effect has a spatial extent, one expects that the mean RT scales with distance from this area. Thus, the distractor location was divided into five distances (dist-0, dist-1, dist-2, dist-3, and dist-4)^1^ from the high probability distractor location. Mean RTs for these conditions are shown in Fig. 3, upper panel. Repeated measures ANOVA on mean RTs showed a significant main effect for *distance*, *F*(4, 92) = 3.12, *p* = .019, partial *η*^*2*^ = .12. Moreover, a linear function was fitted for the data, and its slope was used to clarify whether RT changed with distance. The slope (4.24 ms per display element) was marginally larger than zero, *t*(23) = 1.94, *p* = .064, *d* = 0.56, suggesting a spatial gradient that the suppression effect became smaller when the distractor singleton was present further away from the high probability distractor location. There was no effect on error rates, *F* < 1 (see Fig. 3, upper panel). In the *no-distractor* condition, there was also a significant main effect for *distance*, *F*(4, 92) = 4.43, *p* = .003, partial *η*^*2*^ = .16, but no effect on error rates, *F* < 1.

### Discussion

The current experiment shows that when observers adopt a feature search mode to find the target, statistical regularities regarding the distractor also effect selection. Indeed, for the high probability location, we found that both attentional capture by the distractor and the efficiency of selecting the target were reduced relative to the low probability location. This suggests that statistical regularities regarding the distractor still had an effect on selection even when a feature search mode was adopted.

It is important to note that even though observers must have adopted a feature search mode, there was still a small yet significant capture effect (14 ms) when the distractor appeared at low probability locations. One might argue that the statistical regularities still had an effect while being in feature search because the distraction effect was not completely eliminated. Thus, the ultimate test would be to find a condition in which there is no capture anymore while being in a feature search mode.

## Experiment 2

Even though it is clear that when searching for a specific feature (feature search mode) capture is much smaller (and in most experiments even absent) than when searching for any salient singleton (singleton detection mode), the existence of these separate search modes has not been undisputed. Theeuwes (2004) argues that by introducing different types of elements (circle, triangle, square, and diamond) to force feature search, the search becomes more difficult because none of the elements stand out from the background. If the search becomes more difficult observers use a smaller attentional window (i.e., a more focused search mode) to the find the target, and this smaller window results in a reduction of attentional capture (but see Leber & Egeth, 2006).

Given this reasoning by Theeuwes (2004), one could argue that in Experiment 1 there was still some capture left because the search was not difficult enough to force a small attentional window. Experiment 2 was a 100% replication of Experiment 1 except that observers had to search for a diamond among triangles, squares, and circle. Unlike the target in Experiment 1 (which was a circle), a diamond has rectangular features that resemble the features of squares and triangles, which should make search much harder. We expect that in this condition (which is basically nothing other than a feature search), capture will be eliminated. The question then is when the distraction is completely eliminated, will the statistical regularities regarding the distractor still have an effect?

### Method

Twenty-four adults (19 females, mean age: 20 years) participated in this experiment for monetary compensation. The stimuli, procedure, and experimental design were the same as Experiment 1, except that participants were required to continuously search for the diamond instead of the circle.

### Results and discussion

Twelve percent of trials with larger gaze deviation were later re-tested in a random order. Trials on which the response times (RTs) were larger or smaller than 2.5 standard deviations from the average RT per block per participant (7.3 %) were excluded from analyses.

#### Attentional capture effect

**Fig. 4.**
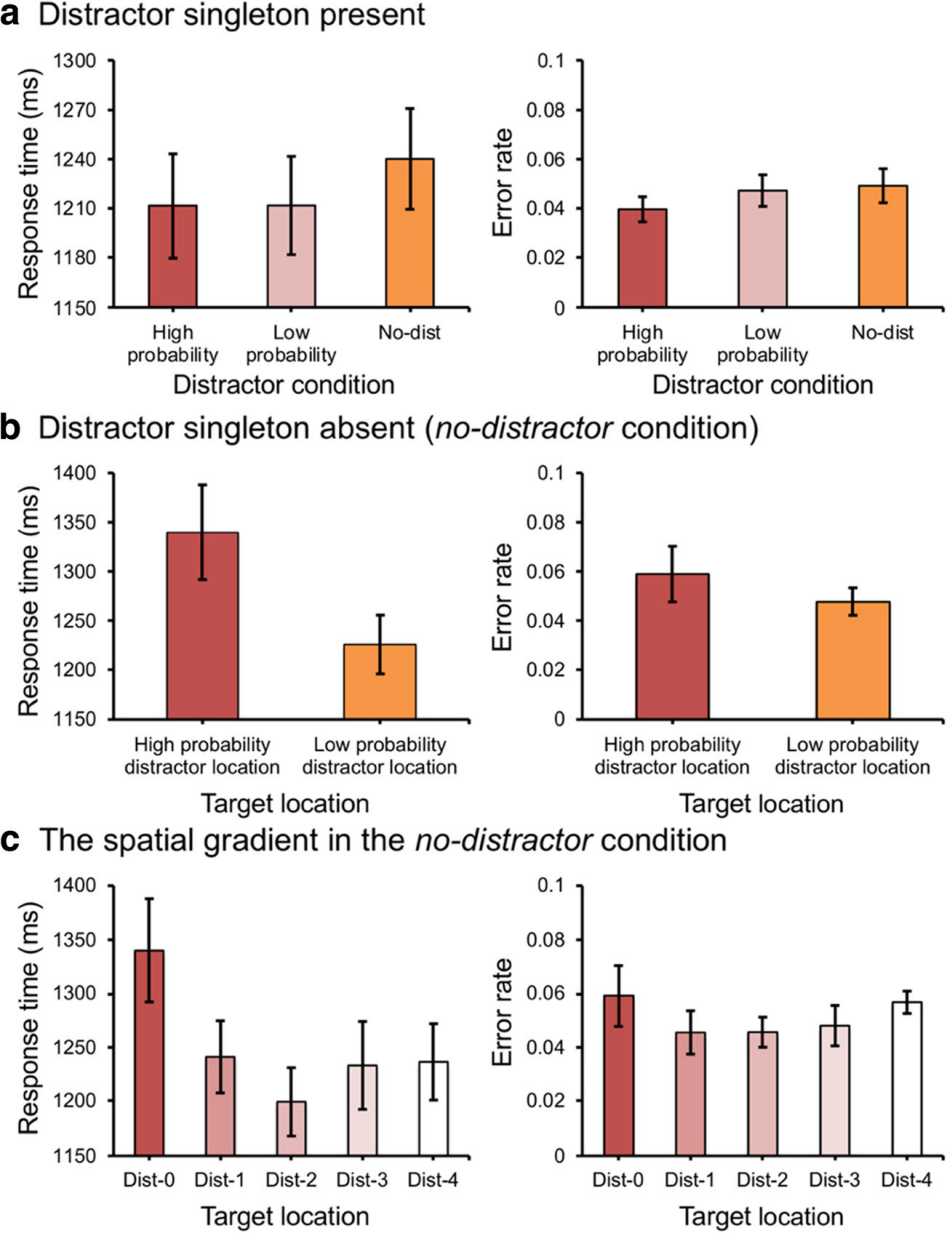
(**A**) The mean response times (RTs) (left panel) and mean error rates (right panel) between different distractor locations in Experiment 2. (**B**) The mean RTs (left panel) and the mean error rates (right panel) in the no-distractor condition in Experiment 2. (**C**) The spatial distribution of suppression effect by the means of RTs (left panel) and error rates (right panel) in the no-distractor condition in Experiment 1. Error bars denote within-subjects 95% confidence intervals

Mean RTs and mean error rates are presented in Fig. 4A. With *distractor condition* (high probability location, low probability location, and no-distractor) as a factor, mean RTs were entered into a repeated measures ANOVA and showed a main effect, *F*(2, 46) = 6.96, *p* = .002, partial *η*^*2*^ = .23. Subsequent planned comparisons showed that instead of attentional capture these effects were reversed; that is the low probability location gave faster mean RTs than the no-distractor location, *t*(23) = 3.27, *p* = .003, *d* = 0.19, and the same for the high probability location relative to the no-distractor location, *t*(23) = 3.30, *p* = .003, *d* = 0.19 (see Gaspelin, Leonard, & Luck, 2015 for similar results). Moreover, the difference between the high and the low probability location was not reliable, *t* < 1.

With *distractor condition* (high probability location, low probability location, and no-distractor) as a factor, mean error rates were entered into a repeated measures ANOVA and showed a main effect, *F*(2, 46) = 3.39, *p* = .042, partial *η*^*2*^ = .13. Subsequent planned comparisons showed that there was no difference between low probability location and no-distractor condition, *t* < 1. The mean error rate was lower when the distractor singleton was present at the high probability location compared to the no-distractor condition, *t*(23) = 2.62, *p* = .015, *d* = 0.32. However, the difference between the high and low probability location was only marginally significant, *t*(23) = 1.81, *p* = .083, *d* = 0.27. This pattern of results suggests that the suppression was partially reflected in error rates when attentional capture effect was completely eliminated.

#### Target at the high probability distractor location

The results above seem to indicate that, if there is no capture, it does not matter much whether a distractor appears much more often in one location than in any of the other locations. However, it is still possible that statistical regularities had an effect on the efficiency of selecting the target. To this end, we analyzed the RTs in the *no-distractor* condition for when the target happened to be present at the high relative to low probability location. Participants were slower when the target was present at the high probability distractor location than when it was present at low probability distractor locations, *t*(23) = 3.48, *p* = .002, *d* = 0.58 (see Fig. 4B, left panel). There was no effect on error rates, *t*(23) = 1.12, *p* = .274, *d* = 0.25 (see Fig. 4B, right panel).

#### The spatial distribution of the suppression effect

Mean RTs and mean error rates are shown in Fig. 4C. Given that there was no suppression effect in the distractor present condition, we only analyzed the suppression effect for the *no-distractor* condition. The distractor location was divided into five distances (dist-0, dist-1, dist-2, dist-3, and dist-4) from the high probability distractor location. Repeated measures ANOVA on mean RTs showed a significant main effect for *distance*, *F*(4, 92) = 4.88, *p* = .001, partial *η*^*2*^ = .18. Again, with increasing distance, the selection of the target became progressively more efficient with a slope of -22.3 ms per display element, which was different from zero, *t*(23) = 2.19, *p* = .039, *d* = 0.63. There was no effect on error rates, *F* < 1.

### Discussion

In the current experiment we completely eliminated attentional capture and still found an effect of statistical regularities regarding the distractor as there was a clear spatial gradient effect of suppression around the high probability location. It is important to realize that this distractor suppression had no measurable effect on the amount of attentional capture as distractors did not cause any RT interference on finding the target, regardless of whether these distractors were presented at a high or a low probability location. Crucially, however, even though the presence of distractors had no measurable RT effect on attentional selection (i.e., they did not cause attentional capture), when the target happened to be presented at the location that was highly likely to contain a distractor, participants were slower to respond to the target relative to all other locations. This finding indicates that the location was suppressed, consistent with Exp. 1 and earlier findings (Wang & Theeuwes, 2018a, b).

One aspect of the results needs further discussion. We observed a reversed capture effect: RTs in the no-distractor condition were slower than when a distractor was present. Further analysis of this reversed capture effect revealed that during the first two blocks of 120 trials there was no distractor suppression. During the subsequent four blocks of 120 trials, the capture effect reversed such that participants were faster in the distractor condition than in the no-distractor condition. One explanation for this effect is that in the distractor condition there is one less location (item) to search as the distractor cannot be the target while in the no-distractor condition all elements need to be searched. It fits with our notion that this type of feature search is difficult and extremely slow (evidenced by the observed RTs of about 1,200 ms in the Experiment) and likely serial in nature. Because of its serial nature, searching one item less (in the distractor condition) will ultimately result in a faster search.

## Experiment 3

In the first two experiments, we introduced statistical regularities regarding the distractor location. Our results show that the location that is highly likely to contain a distractor is suppressed relative to all other locations. One question that these studies cannot answer is whether this location-specific suppression is modulated by introducing statistical regularities regarding the feature of the distractor. Indeed, in our studies the distractor was equally likely to contain either a red or a green distractor randomized across trials. The question is whether introducing one color more often than the other color would modulate the spatial suppression.

This manipulation is relevant because recently Gaspelin and Luck (2018a) showed in their studies that distractor suppression does not work for just any salient singleton but rather depends on the exact feature value (e.g., red vs. green). Experiments 3 and 4 were set out to test whether in addition to statistical learning-induced spatial suppression, statistical regularities regarding the feature of the distractor would further modulate suppression. In Experiment 3, we systematically presented the distractor in one color much more often than in the other color. If the feature value of the distractor plays a role, we expect to see a stronger suppression for the frequent color than for the less frequent color. If the suppression is fully location-based we expect to see no difference between these conditions.

### Method

Twenty-four adults (20 females, mean age: 19.8 years) participated in the present experiment for monetary compensation. The stimuli, procedure, and experimental design were the same as Experiment 1, except that the chance of containing the green distractor singleton and that of containing the red distractor singleton were not equal anymore. In *distractor singleton present* trials, one of these colors had a high proportion of 80% (high probability distractor color), and the other color had a low proportion of 20% (low probability distractor color). The high probability distractor color was unchanged for each participant and was counterbalanced across participants.

### Results and discussion

Eight percent of trials with larger gaze deviation were later re-tested in a random order. Trials on which the RTs were larger or smaller than 2.5 standard deviations from the average RT per block per participant (2.9%) were excluded from analyses.

**Fig. 5.**
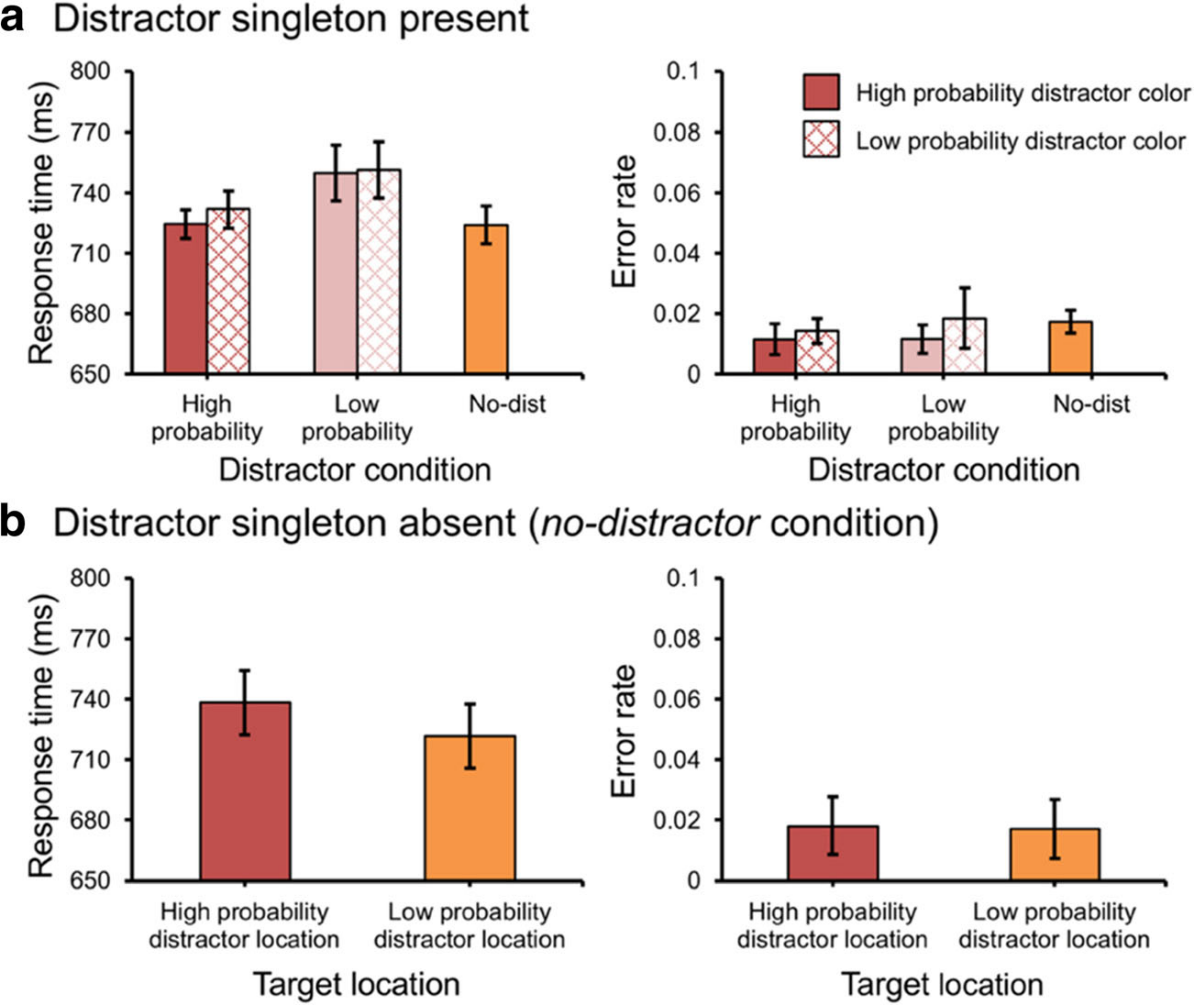
(**A**) The mean response times (RTs) (left panel) and mean error rates (right panel) between different distractor locations and different distractor colors in Experiment 3. (**B**) The mean RTs (left panel) and the mean error rates (right panel) in the no-distractor condition in Experiment 3. Error bars denote within-subjects 95% confidence intervals

To explore whether the location-based suppression effect was affected by the predictability of the feature value (i.e., the distractor color), we conducted an ANOVA on mean RTs with *distractor location* (high vs. low probability location) and *distractor feature* (high vs. low probability color) as factors. There was a main effect for *distractor location*, *F*(1, 23) = 19.1, *p* < .001, partial *η*^*2*^ = .45, but not for *distractor feature*, *F*(1, 23) = 1.18, *p* = .29, partial *η*^*2*^ = .05. Importantly, there was no interaction, *F* < 1. There was no effect on error rates, all *p*s > 0.14 (see Fig. 5). These findings indicate that the feature value plays no role in the spatial suppression of the high probability location.

When we collapsed over feature value, we basically found the same results as in Experiment 1: We observed a significant attentional capture effect for a distractor present at the low probability location, *t*(23) = 3.65, *p* = .001, *d* = 0.13, but not for a distractor present at the high probability location, *t* < 1. The difference between the high and low probability location was reliable, *t*(23) = 4.32, *p* < .001, *d* = 0.15. There was no effect on error rates, *F*(2, 46) = 2.82, *p* = .07, partial *η*^*2*^ = .11. For the *no-distractor* condition, we found that participants were slower when the target was present at the high versus the low probability distractor location, *t*(23) = 2.2, *p* = .038, *d* = 0.17. There was no effect on error rates, *t* < 1. Also, as in Experiment 1, there was an effect of *distance* on RTs from the high probability location. The slope (5.88 ms per display element) was larger than zero, *t*(23) = 3.82, *p* < .001, *d* = 1.1, indicating evidence for a spatial suppression gradient. Moreover, this effect reversed in the *no-distractor* condition with a slope of -4.79 ms per display element, which was marginally different from zero, *t*(23) = 1.87, *p* = .074, *d* = 0.54. This latter effect suggests the spatial gradient of the suppression effect is independent of whether a distractor is present or not. There was no effect on error rates, both *t*s < 1. The current experiment shows that the predictability of the feature that needed to be suppressed plays no role in the amount of suppression. Unlike the suppression effects reported by Gaspelin and Luck (2018a), the suppression effects reported here seem to be only location specific.

## Experiment 4

One may question whether Experiment 3 is a fair test of feature suppression as the color of target and distractor constantly and randomly swap. Because the colors swap, on one trial, the to-be-suppressed distractor feature may be the target feature on the next trial. Thus, in Experiment 3 these colors were not consistently associated with distractor processing only, and therefore there may have been little room for feature suppression to occur. To test this further, in Experiment 4 we present all elements in gray except for the distractor, which could appear in red or green. Again, balanced across participants one color was present much more often (80% of the trials) than the other color (20%).

### Method

Twenty-four adults (22 females, mean age: 19.1 years) participated in the present experiment for monetary compensation. The stimuli, procedure, and experimental design were the same as in Experiment 3, except that all the display elements (except for the distractor) were gray.

### Results and discussion

Six percent of trials with larger gaze deviation were later re-tested in a random order. Trials on which the RTs were larger or smaller than 2.5 standard deviations from the average RT per block per participant (2.6%) were excluded from analyses.

**Fig. 6.**
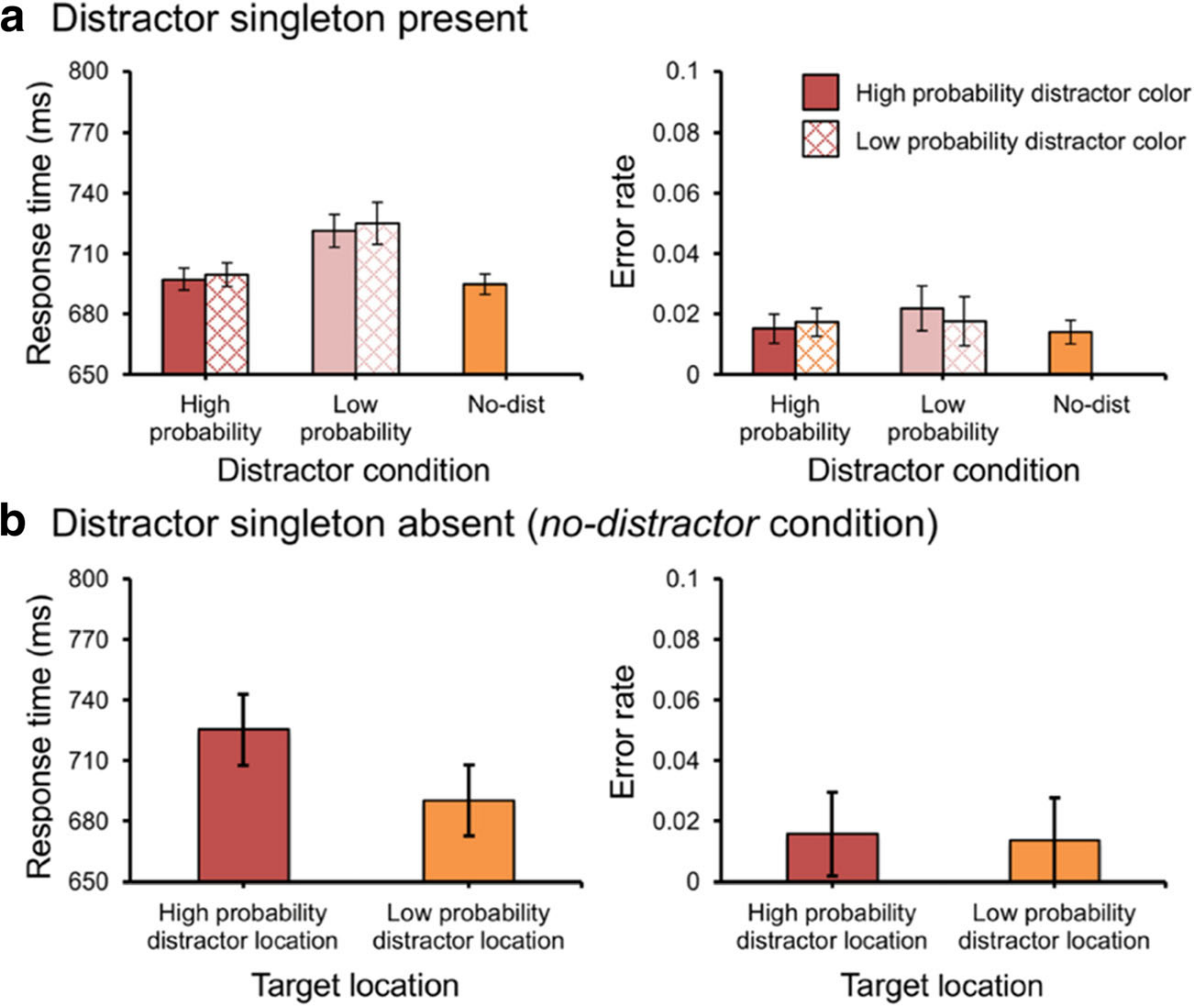
(**A**) The mean response times (RTs) (left panel) and mean error rates (right panel) between different distractor locations and different distractor colors in Experiment 4. (**B**) The mean RTs (left panel) and the mean error rates (right panel) in the no-distractor condition in Experiment 4. Error bars denote within-subjects 95% confidence intervals

Again, to explore whether the location-based suppression effect was affected by the predictability of the feature value (i.e., the distractor color), we conducted an ANOVA on mean RTs with *distractor location* (high vs. low probability location) and *distractor feature* (high vs. low probability color) as factors. There was a main effect for *distractor location*, *F*(1, 23) = 50.76, *p* < .001, partial *η*^*2*^ = .69, but not for *distractor feature*, *F*(1, 23) = 1.44, *p* = .24, partial *η*^*2*^ = .06. Importantly, there was no interaction, *F* < 1. There was no effect on error rates, all *p*s > 0.213 (see Fig. 6). These findings indicate that the feature value plays no role in the spatial suppression of the high probability location (Fig. 6).

When we collapsed over feature value, we basically found the same results as in the previous experiments: We observed a significant attentional capture effect for a distractor present at low probability locations, *t*(23) = 8.36, *p* < .001, *d* = 0.28, but not for a distractor present at the high probability location, *t*(23) = 1.17, *p* = .254, *d* = 0.03. The difference between the high and low probability location was reliable, *t*(23) = 7.01, *p* < .001, *d* = 0.24. For the error rates, there only existed an attentional capture effect for a distractor present at low probability locations, *t*(23) = 2.08, *p* = .048, *d* = 0.34. In the *no-distractor* condition, we found that participants were slower when the target was present at the high versus the low probability distractor location, *t*(23) = 4.27, *p* < .001, *d* = 0.33. There was no effect on error rates, *t* < 1. Also, as in Experiments 1 and 2, there was an effect of *distance* on RTs from the high probability location. The slope (5.73 ms per display element) was larger than zero, *t*(23) = 3.28, *p* < .001, *d* = 0.95, indicating evidence for a spatial suppression gradient. Moreover, this effect reversed in the *no-distractor* condition with a slope of -9.26 ms per display element, which was different from zero, *t*(23) = 3.03, *p* = .006, *d* = 0.87. This latter effect suggests the spatial gradient of the suppression effect is independent of whether a distractor is present or not. There was no effect on error rates, both *t*s < 1.

#### Comparing capture effects in singleton detection and feature search modes

In Wang and Theeuwes (2018a), observers used a singleton detection mode and the capture effect was 117 ms. In the current study these capture effects were 14 ms (Experiment 1), 26 ms (Experiment 3), and 28 ms (Experiment 4). The capture effect is the difference in RT between a distractor in the low probability location relative to the no-distractor condition. This represents an adequate estimation of the amount of capture because the low probability location is not (or only slightly) impacted by the spatial regularity manipulation (while the high probability location is strongly affected). The difference in capture between singleton detection and feature search modes were reliable in Experiment 1, *t*(23) = 10.77, *p* < .001, *d* = 3.2, in Experiment 3, *t*(23) = 7.93, *p* < .001, *d* = 2.39, and in Experiment 4, *t*(23) = 9.63, *p* < .001, *d* = 2.87.

#### Comparing suppression effect between singleton detection and feature search modes

In order to estimate the suppression effect in singleton detection and feature search modes, we calculated the difference in RT between the high and low probability locations of Wang and Theeuwes (2018a) and the present study. The suppression effect was much larger in singleton detection mode (67 ms) than in the current study (Experiment 1: 14 ms, Experiment 3: 23 ms, and Experiment 4: 24 ms). These differences were reliable in Experiment 1, *t*(23) = 6.52, *p* < .001, *d* = 2, Experiment 3, *t*(23) = 4.86, *p* < .001, *d* = 1.51, and Experiment 4, *t*(23) = 5.26, *p* < .001, *d* = 1.68.

## General discussion

In the present study, observers had to use the so-called *feature search mode* to find a specific shape (e.g., a circle) among a heterogeneous set of distractor shapes. Instead of looking for singletons, observers had to use a shape template to find the target (Bacon & Egeth, 1994). There is consensus that when observers use the feature search mode, they are able to exert top-down control to avert attentional capture by the salient singleton (Bacon & Egeth, 1994; Leber & Egeth, 2006). Specifically, it has been argued that *“subjects using this mode should not be susceptible to capture by stimuli not matching the attentional set*” (Leber & Egeth, 2006; p. 133). Because the distractor singleton does not match observers’ attentional “feature” set, it assumed that observers should be able to completely ignore the distractor. If this is the case, then statistical regularities that we introduced should not have been useful, because top-down control should have made it possible to ignore distractors regardless of their (high vs. low probability) locations. The current results indicate the opposite: similar to Wang and Theeuwes (2018a, b) we show that both attentional capture by the distractors (Exps. 1, 3, and 4) and the efficiency of selecting the target (Exps. 1, 2, 3, and 4) were impacted by these statistical regularities.

Alternatively, instead of simply ignoring the salient distractor, it has been argued that in the feature search mode, the presence of a distractor does affect processing in the sense that observers suppress the location containing the salient distractor. Indeed, recent studies have demonstrated that during feature search, the presence of an irrelevant singleton elicits a PD component, representing the neural maker of suppression (Burra & Kerzel, 2014; Eimer & Kiss, 2008; Feldmann-Wüstefeld et al., 2015; Sawaki & Luck, 2010, 2013). For example, Sawaki and Luck (2010) argued that salient distractors generate an attention capture signal. Yet, by choosing the feature search mode, participants are able to actively suppress the location of the salient distractors, such that the deployment of attention to these distractors can be avoided (see also Gaspelin et al. 2015). In this respect, the current findings show that through statistical learning, suppression due to feature search is more pronounced (less capture) for those locations that are highly likely to contain a distractor than for all other locations. This suppression also results in less efficient selection of the target when it happens to be presented at the high probability location. In their original conception of the signal suppression hypothesis, Sawaki and Luck (2010, 2013) argued that this suppression was the result an active, top-down process while we argue here that this suppression is the result of statistical learning (see Wang & Theeuwes, 2018b). Note, however, that in more recent conceptions of the signal suppression hypothesis, it is conceived that suppression can be the result of selection history and/or the result of active, top-down suppression (Gaspelin & Luck, 2018b).

The current findings are consistent with our previous finding (Wang & Theeuwes, 2018a, b). As before we show that attentional capture by a salient singleton present at locations that are more probable to contain a distractor is reduced relative to capture by a singleton present at locations that are less probable to have a distractor. Also, if a target singleton happened to be present at this location, its selection was less efficient than when the target singleton was present at other locations. Crucially, we again show a spatial gradient of suppression. We found that in the no-distractor condition the selection efficiency scales with distance from the probability location such that with increased distance, the selection of the target became progressively more efficient. The reverse was found for the distractor condition: Here the distractor interference effect became progressively stronger with the increase in the distance from the high probability location. Both effects point to a spatial suppression effect with a spatial gradient surrounding the location.

Experiments 3 and 4 show that the predictability of the feature that needs to be suppressed played no role in the amount of suppression. This implies that suppression induced by learned statistical regularities regarding the location of the distractor are not feature-specific. It is important to note that these findings have no bearing on previous studies using other paradigms that unequivocally demonstrated feature-specific suppression (e.g., Gaspelin & Luck, 2018a; Sawaki & Luck, 2010). The current findings just show that statistical learning about specific features plays no role in suppression above and beyond suppression based on location.

The current experiments also shed some light on the discussion regarding the assumed search modes referred to as feature search and singleton detection modes. We have shown in three of the four experiments that participants that are in a feature search mode searching for a specific feature (a circle between rectangular other elements like diamonds, squares) still may show some relatively small capture effect. Previous studies employing feature search have also reported capture effects (Graves & Egeth, 2016; Theeuwes, 2004; Vatterott & Vecera, 2012; Zehetleitner et al., 2012). However, our Experiment 2 in which participants searched for a diamond instead of a circle between rectangular non-targets showed much slower search times and no capture, which seem to be in line with earlier claims that these search modes represent nothing more than a gradual difference between a parallel and (clump-wise) serial search (Theeuwes, 2004). The current paper is not about the difference between feature search and singleton detection mode, but instead only demonstrates that even when participants search for a specific target feature, statistical regularities about the distractor location play a role. In experiments in which there was capture (Exps. 1, 3, and 4), these statistical regularities reduced the capture for locations that were highly likely to contain a distractor. In Experiment 2 in which there was no capture, the target selecting at the location that was highly likely to contain a distractor was less efficient, suggesting that even without attentional capture by the distractors, the location that was highly likely to contain a distractor was suppressed relative to all other locations.

With respect to the discussion of feature search versus singleton detection modes, it is clear that when adopting singleton detection mode capture effects are much larger (Wang & Theeuwes, 2018a for example reported 117 ms) than when adopting feature search mode (current capture effects were 14 ms (Exp. 1), 0 ms (Exp. 2) 26 ms (Exp. 3), 28 ms (Exp. 4)). Even though using the feature search mode dramatically reduces the size of capture, the crucial point to make is that in three out of the four experiments capture was not eliminated. The current most accepted notion is that when participants strategically choose the so-called feature search mode they are able to impose top-down selectively. In this mode, participants should no longer be susceptible to capture by stimuli that do not match their attentional set (Leber & Egeth, 2006). However, according to Theeuwes (2004), feature search has nothing to do with strategically choosing another search mode; instead, while searching for a specific target feature, search becomes much harder, forcing participants to open a small attentional window encompassing only one or a few items at a time and move clump-wise serially around the display until the target is found (see also Theeuwes, 1994). Only inside this window is there salience-based competition, implying that when a distractor is outside the window it does not affect capture anymore. Previous studies have provided some evidence for this notion. For example, Belopolsky, Zwaan, Theeuwes and Kramer (2007) manipulated the size of the attentional window and showed that only when the window was set broad, did salient distractors capture attention (see also Belopolsky & Theeuwes, 2010). The current findings are very much in line with this notion as we found no capture anymore when the search task was very difficult (Exp. 2) and small capture effects in Exps. 1, 3, and 4 when the search was relatively easy. The bottom-line is that feature search reduces capture dramatically relative to singleton search, but whether this is due to a strategic top-down set (Leber & Egeth, 2006; Sawaki & Luck, 2010) or whether it is induced by the properties of the display (i.e., whether search is difficult or not) is still a matter of debate.

In sum, even when observers search for a specific feature (the so-called feature search mode) in which top-down control should prevent the selection of the distractor, statistical regularities regarding the distractor location cause an attentional bias affecting the amount of attentional capture by the distractor and the efficiency of selection of the target. Statistical regularities regarding the feature value of the distractor (its color) had no effect on the amount of capture or the efficiency of selection. We conclude that regardless of whether observers search for a singleton or for a specific feature value, statistical regularities cause passive lingering biases of attention such that weights within the priority map are changed, causing the location that is likely to contain a distractor to compete less for attention than all other locations.

### Author notes

This research was supported by a European Research Council (ERC) advanced grant [ERC-2012-AdG-323413] to JT and a China Scholarship Council (CSC) scholarship [201508330313] to BW. The authors would like to thank Nick Gaspelin and two anonymous reviewers for their excellent suggestions and comments.

## Footnotes

1 Dist-0 location means the high probability distractor location; Dist-1 location means that the distractor was present next to the high probability distractor location (45° polar angle); dist-2 means 90° from the high probability distractor location; dist-3 location means 135° from the high probability distractor location; dist-4 location means 180° from the high probability distractor location.
